# EvoAI enables extreme compression and reconstruction of the protein sequence space

**DOI:** 10.21203/rs.3.rs-3930833/v1

**Published:** 2024-02-23

**Authors:** Shuyi Zhang, Ziyuan Ma, Wenjie Li, Yunhao Shen, Yunxin Xu, Gengjiang Liu, Jiamin Chang, Zeju Li, Hong Qin, Boxue Tian, Haipeng Gong, David Liu, B Thuronyi, Christopher Voigt

**Affiliations:** Tsinghua University; Tsinghua University; Tsinghua University; Tsinghua University; Tsinghua University; Tsinghua University; Tsinghua University; Tsinghua University; Tsinghua University; Tsinghua University; Tsinghua University; Broad Institute; Williams College; Massachusetts Institute of Technology

## Abstract

Designing proteins with improved functions requires a deep understanding of how sequence and function are related, a vast space that is hard to explore. The ability to efficiently compress this space by identifying functionally important features is extremely valuable. Here, we first establish a method called EvoScan to comprehensively segment and scan the high-fitness sequence space to obtain anchor points that capture its essential features, especially in high dimensions. Our approach is compatible with any biomolecular function that can be coupled to a transcriptional output. We then develop deep learning and large language models to accurately reconstruct the space from these anchors, allowing computational prediction of novel, highly fit sequences without prior homology-derived or structural information. We apply this hybrid experimental-computational method, which we call EvoAI, to a repressor protein and find that only 82 anchors are sufficient to compress the high-fitness sequence space with a compression ratio of 10^48^. The extreme compressibility of the space informs both applied biomolecular design and understanding of natural evolution.

Protein engineering and design can create proteins with optimized functions for various applications in biotechnology, medicine, and synthetic biology^1–3^. The fundamental challenge of protein engineering is to understand and manipulate the protein fitness landscape, which is a high-dimensional and complex space that contains a vast number of possible sequences and functions^4,5^.

Although there have been considerable attempts over the past several decades to search this space for high-fitness sequences, we have only scratched the surface of understanding the rules and features of the space^6–9^.

Experimental methods using directed evolution techniques, such as deep mutational scanning^10,11^, site-saturated mutagenesis^12,13^, and random library construction^14,15^, can provide valuable information, but they are laborious and time-consuming to scale up and typically must trade off accuracy and precision with sequence space coverage. These experimental methods are also usually restricted to low-dimensional mutations that do not take into account the natural selection pressure that shapes the protein fitness landscape in high-dimensional space. Advanced directed evolution tools that support the necessary scale, such as phage-assisted continuous evolution (PACE)^16,17^ or OrthoRep^18^, provide information primarily about trajectories that lead to high-fitness variants, which is insufficient to model the fitness landscape in its entirety. Computational methods, such as structure or sequence-based modeling of the protein fitness landscape^19–25^, can evaluate larger sequence spaces but are limited by the availability and quality of training data, especially for proteins with few homologs or no structure information. These computational methods also typically do not account for other biological factors that affect the protein function, such as *in vivo* interactions or post-translational modifications.

An ideal approach to understanding and navigating this space for design and engineering purposes would use comprehensive high-throughput experimental data to inform efficient computational models. It was shown that high-throughput short sequencing data from directed evolution experiments can enable machine learning methods to reconstruct the full-length genotype and identify high-fitness variants^26^. Furthermore, it has been demonstrated that deep learning models for protein design can benefit from even a limited number of functionally characterized variants^27^. A recent work demonstrated that the protein fitness landscape is rugged with many local peaks but still easily navigable^28^. We view these functional variants or local peaks as key “anchor” points that capture the features of high-fitness genotype space. We hypothesize that the design space for high-fitness genotypes can be effectively compressed by identifying a sufficient number of these “anchor” points to capture all the essential features, which can then instruct deep learning models to reconstruct and explore the whole space. However, no existing method can generate these anchors in a rapid and comprehensive way, especially for anchors from the high-dimensional space. Such a method would need to capture functional information about variants evenly distributed across protein sequence space in a very high throughput manner.

Here, we present EvoAI, a novel approach to empirically interrogate, then model, compress, and reconstruct, the sequence space. Our approach combines high-throughput experimental evolution and computational methods to capture and learn from the essential features of the space. We first developed an evolutionary scanning method that adapts phage-assisted non-continuous evolution (PANCE)^17^ by incorporating a segmented mutagenesis system based on EvolvR^29^. Compared to traditional methods, this method enabled rapid and thorough evolutionary scanning from low to high dimensions and captured valuable fitness anchors. We then developed a deep learning and large language model to reconstruct the sequence space from these anchors and design new proteins with more than 10-fold improved activity compared to wild-type. For a repressor protein, we demonstrated that this vast design space can be extremely compressed by a factor of 10^48^ to 82 points.

## Results

### The evolutionary scanning method

**Figure 1:**
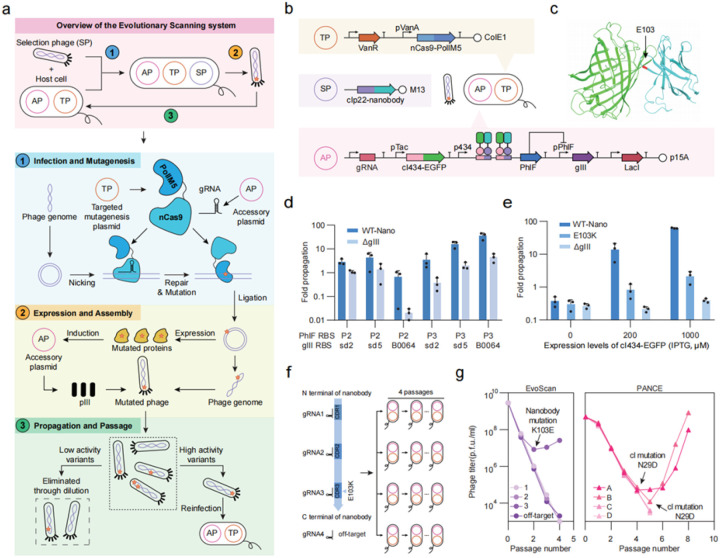
EvoScan scheme, development, and validation on a protein-protein interaction evolution. (a) Overview of the Evolution Scanning system. (b) Testing scheme for the EvoScan system on EGFP-nanobody interaction. (c) Predicted interaction between EGFP and nanobody. The structures of EGFP and nanobody were predicted by Alphafold2, and the interaction was modeled by ZDOCK (method). The position of the E103 site is labeled in red. (d) Propagation assays of combinatorial AP designs. Several ribosome binding sites (RBS) were tested, including: P2 and P3 for PhlF, and sd2, sd5 and B0064 for gIII. (e) Propagation assays of WT-Nano phage, the E103K mutant phage, and empty phage (ΔgIII) under different concentrations of IPTG (0, 200 μM and 1 mM) to control cI434-EGFP expression levels. (f) EvoScan of the EGFP nanobody. Three different gRNAs targeted different CDR regions of the E103K nanobody. An off-target gRNA with no target sequence in the nanobody was used as a control group. (g) Phage propagation and mutations of EvoScan and PANCE for EGFP nanobody. Initial titers of phage E103K were 3×10^9^ p.f.u. /ml for EvoScan and 5×10^8^ p.f.u. /ml for PANCE. Dilution factor was 100 for each passage. Sequences of sgRNA and genetic parts are provided in Supplementary Table 4 and Table 5. Data are mean ± SD of three experiments, except for phage titers.

The M13 bacteriophage has a single-stranded DNA genome, but it generates a double-stranded form after infecting the host cell^30^ (Fig. 1a). We reasoned that this should allow the targeted CRISPR-guided DNA polymerase mutagenesis system (TP) to introduce mutations into the M13 phage genome for selection and evolution^29^. Here, the expression of the nCas9-PolI complex was controlled by the vanillic acid induced VanR-pVanA expression system that has a large induction fold change and low background expression, and is suitable for expressing large and highly toxic proteins^31,32^. The evolution target was inserted into the M13 genome in place of gIII (the major coat protein of M13) to generate the selection phage (SP). The accessory plasmid (AP) expresses guide RNAs (gRNAs) that target different regions of the gene of interest for mutagenesis. The AP also contains gIII under the control of a genetic circuit that links the function of the gene of interest to the expression of gIII. This allows the selection of phages with improved and high-fitness protein function during phage propagation, while phages with non-functional genes are eliminated after dilution (Fig. 1a). We named this system EvoScan (Evolutionary Scanning).

EvoScan can explore specific regions of the fitness landscape to generate valuable anchors. These anchors are obtained by using different gRNAs to divide the target gene into defined segments, thus reducing the dimensionality of the fitness space. Moreover, the combination of different gRNAs through serial propagation on host cells bearing different APs enables the scanning and identification of anchors in higher dimensions, which can capture more details of the protein sequence space. To investigate and scan the protein sequence space, we validated and used this system to study three proteins with diverse functions: an EGFP-specific nanobody for protein-protein interaction; SARS-CoV-2 M^pro^ and its inhibitors for protein-ligand interaction; and AmeR and its DNA operator for protein-nucleic acid interaction.

### Validation of EvoScan and rapid identification of anchors in nanobody

To validate EvoScan and apply this system to proteins involved in protein-protein interaction, we chose antigen-antibody interaction, in this case, EGFP and its cognate nanobody^33^. We first established a reverse two-hybrid system (RTHS) that coupled the nanobody-EGFP interaction to the expression of gIII. We fused EGFP to the cI434 repressor, and its nanobody to cIp22, which can interact with cI434 but not with itself^34^. The gene encoding nanobody-cIp22 was inserted on phage to replace gIII. The gene encoding EGFP-cI434 was integrated on the AP and transformed into *E. coli*. After phage infection, interaction between EGFP and nanobody will enable the interaction between cI434 and cIp22 to form a tetramer complex and inhibit the p434 promoter (Fig. 1b, 1c). In the AP, a transcriptional repressor PhlF was placed downstream of the p434 promoter, and gIII was placed under the control of the pPhlF promoter, such that interaction between EGFP and the nanobody will eventually induce the expression of gIII and allow phage propagation (Fig. 1b). We tested several combinations of ribosome binding sites and chose P3 RBS for PhlF and B0064 for gIII (Fig. 1d). This circuit propagated phage carrying EGFP nanobody while limiting the propagation of empty phage.

To test whether EvoScan could quickly identify a fitness-increasing protein variant “anchor” site, we artificially disrupted the interaction between EGFP and nanobody by introducing the E103K mutation in the CDR3 of the nanobody, which is essential for binding to its target (Fig. 1c, Fig. 1e). We designed four different gRNAs targeting different segments of the nanobody gene, with gRNA3 designed to target the segment containing the E103K mutation site of the nanobody (Fig. 1f). After two passages in EvoScan, we observed that only the group with gRNA3 targeting the E103K segment showed increased phage titer, while the other three groups all decreased (Fig. 1g). Sequencing results of the phage supernatant confirmed that in the gRNA3 group, the E103K mutation had reverted back to glutamate. This validated that EvoScan can successfully and efficiently identify anchors that play important roles in protein function.

For comparison, we also implemented a traditional phage-assisted non-continuous evolution system (PANCE) using the same E103K phage (Extended Data Fig. 1a). The two systems differed only in the use of targeted (EvoScan, TP) or non-targeted (PANCE, MP6) mutagenesis. After 8 passages in PANCE, no consensus mutations were found in the nanobody gene. Interestingly, a N29D single mutation appeared on cIp22 (Extended Data Fig. 1b, Fig. 1g), which disrupted the selection pressure on nanobody function due to the strong self-interaction between the two cI repressors (Extended Data Fig. 1c, 1d). These results further demonstrated that EvoScan can rapidly guide the evolution for precise searching of target proteins, even in the context of a more likely background mutation that could interfere with the desired evolution process.

### Thorough identification of anchors reveals novel M^pro^ drug resistant variants

We next applied EvoScan to investigate protein-ligand interaction. In this case, we chose M^pro^, a crucial protease in the SARS-CoV-2 virus^35,36^. Several M^pro^ inhibitors have been developed and used to treat COVID-19 patients, such as GC376^37^ and PF-07321332^38^, which is a key component of Paxlovid. However, the rapid mutation of SARS-CoV-2 may reduce or even eliminate the efficacy of these drugs. Previous studies have identified mutational hotspots for drug resistance but have not comprehensively profiled the M^pro^ drug resistance fitness landscape^39,40^. It is important to thoroughly study possible escape mechanisms of M^pro^ in order to inform future drug development efforts. Here, we used EvoScan to systematically identify and extract key anchors from different regions of M^pro^ that affect its interaction with small molecule inhibitors.

**Figure 2:**
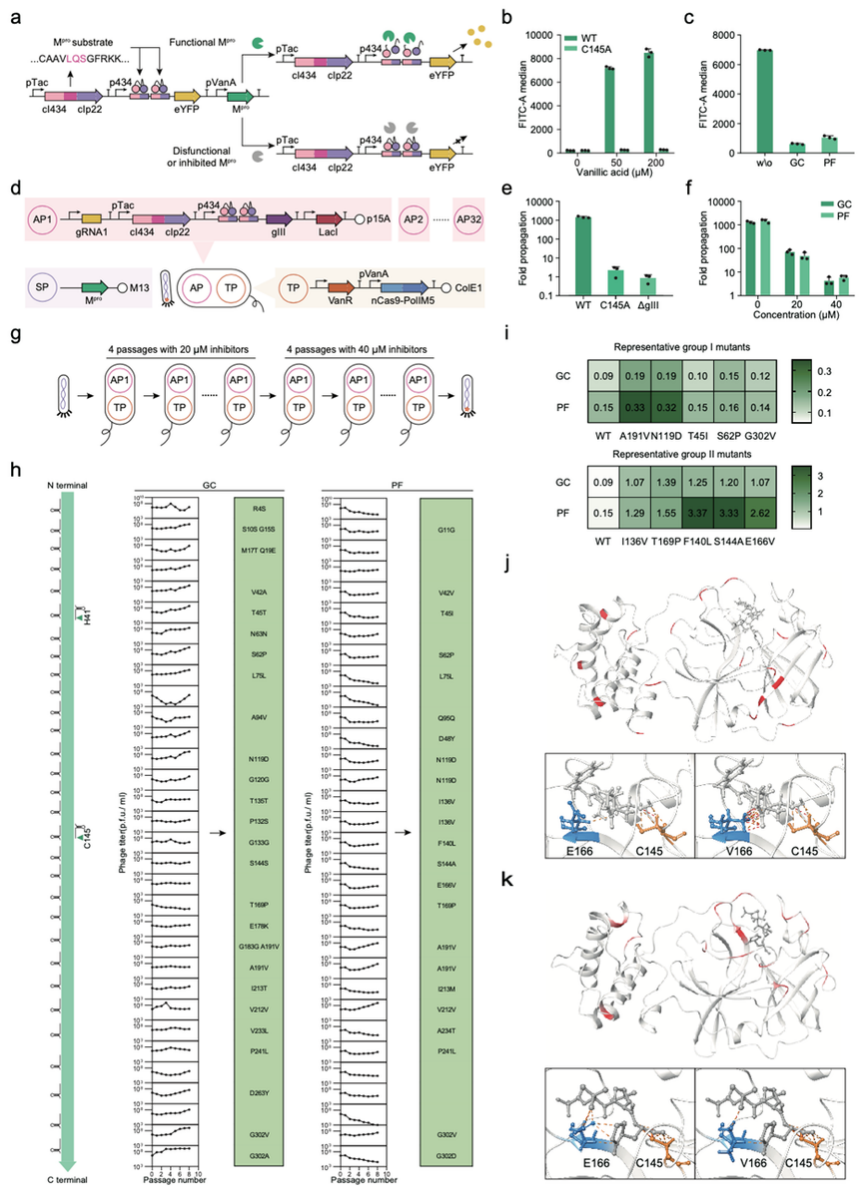
Thorough segment scanning for protein-ligand interaction evolution. (a) A schematic of the M^pro^ activity fluorescence reporter system. The M^pro^ substrate peptide CAAVLQSGFRKK was cloned into the linker between cI434 and cIp22 so that protease activity controlled the output (eYFP) from the p434 promoter. (b) Flow cytometry assays for M^pro^ and the C145A mutant under different inducer concentrations (0, 50 and 200 μM vanillic acid, and 200 μM IPTG). (c) Flow cytometry assays for M^pro^ activity in the presence of 50 μM inhibitors (without inhibitor (w/o), GC376 (GC) and PF-07321332 (PF)). The concentration of IPTG was 200 μM and the concentration of vanillic acid was 100 μM. (d) Genetic circuit design for M^pro^ evolution. 32 different APs carrying 32 different gRNAs tiling the M^pro^ gene sequence were designed. (e) Phage propagation assays for M^pro^, the C145A mutant, and the empty phage at 1 mM IPTG. (f) Phage propagation assays in the presence of 0, 20, or 40 μM inhibitors. (g) Schematic diagram of the M^pro^ EvoScan process. (h) EvoScan of M^pro^ using two different inhibitors and 32 different gRNAs. The initial titer of M^pro^ phage was 5×10^6^ p.f.u. /ml. (i) The resistance index (RI) against GC376 (50 μM) and PF-07321332 (50 μM) of different variants. (j, k) Crystal structure of WT M^pro^ interacting with GC376 (j, PDB ID: 7CB7) and PF-07321332 (k, PDB ID: 7VLO). Mutated residue sites obtained in EvoScan are highlighted in red. Interactions between key residues and the ligand are indicated with dash lines in the enlarged figures. Sequences of gRNAs and genetic parts are provided in Supplementary Table 4 and Table 5. Data are mean ± SD of three experiments, except for phage titers.

To couple the protease activity of M^pro^ to the expression of downstream reporter genes, we fused the two cI repressors, cI434 and cIp22, with a linker that contains the specific sequence motif recognized by M^pro^, such that only functional M^pro^ will cleave and deactivate the fused cI repressor (Fig. 2a). We also used a previously reported inactive M^pro^ mutant (C145A) to validate this system^35,36^. Our results demonstrated that this selection circuit can accurately and sensitively report on M^pro^ activity and the inhibition efficiency of small molecules (Fig. 2b, 2c). In addition, we found that this genetic circuit can be used for proteases from other viruses such as HCV (Extended Data Fig. 2a, 2b), demonstrating its robustness and broad applications. Compared to previously reported selections used for protease evolution in PACE^41–43^, our circuit represents an alternative and improved strategy with better response properties (Extended Data Fig. 2c, 2d).

We then used our genetic circuit to couple the cleavage activity of M^pro^, encoded on SP, to the expression of gIII, which was controlled by the p434 promoter (Fig. 2d). This circuit enables selection for M^pro^ variants that can escape inhibition by small molecules. Our results showed that wild-type M^pro^ supported robust phage propagation, while the C145A mutant behaved like empty phage (Fig. 2e, Extended Data Fig. 2e). We tested phage propagation at various concentrations of the inhibitors GC376 and PF-07321332, and selected 20 μM as the initial concentration for evolution (Fig. 2f, Extended Data Fig. 2f).

We designed 32 different gRNAs to systematically cover the M^pro^ gene and performed EvoScan with two inhibitors (Fig. 2g, 2h). Surprisingly, we found that escaping mutations can occur across the whole M^pro^ gene (Fig. 2h). Some of these mutations, such as F140L, E166V, and S144A, have also been reported in previous studies on drug resistance against PF-07321332^44,45^, proving the effectiveness and reliability of our system. Most other mutations were not observed in previous works, demonstrating that EvoScan can successfully identify novel key mutations. We also identified conserved mutation sites for both inhibitors, such as S62, L75, N119, S144, T169, A191, P241, and G302 (Fig. 2h). Interestingly, we observed that the phage propagation trajectories of the 32 segments targeted for mutagenesis varied during the evolution process, and more than 10 segments showed no overall enrichment during serial passaging, suggesting that mutations within each of these segments taken individually cannot enable drug resistance, which may serve as regions for future drug development studies (Fig. 2h).

We further verified the ability of these mutations to confer inhibition resistance (Fig. 2i, Extended Data Fig. 2g, 2h). Nearly all of these mutations showed increased resistance against inhibitors compared to wild-type M^pro^. In group I mutations, we found that A191V had a strong resistance effect against both inhibitors, while N119D had a moderate resistance effect, and other mutations had relatively weak resistance effects on their specific inhibitors. Strikingly, we found a set of group II mutations (such as E166K), of which the enzyme activities were even improved by inhibitors (Fig. 2i). Similar to a previously reported mechanism where GC376 increased the catalytic activity of M^pro^ mutants^46^, E166K has a different interaction with the inhibitors compared to WT, which may then improve the dimerization of M^pro^ and thus the enzyme activity (Fig. 2j, Fig. 2k). The same phenomenon was observed with other mutations such as I136V, T169P, F140L, and S144A. However, how these mutation sites increase the enzyme activities when inhibitors were added is not clear, as they are located far from the active pocket.

As a comparison, we also evolved M^pro^ using the PANCE system (Extended Data Fig. 3a, 3b). With only the mutagenesis method changed, M^pro^ SP failed to accumulate any consensus mutations after 36 passages. After 96 passages, 4 dominant variants with escaping abilities emerged in the four groups in total (Extended Data Fig. 3c-f). All these variants have the N119D or A191V mutation, which appeared after only 8 passages in EvoScan. These results further showed that EvoScan can effectively explore protein-ligand interaction and identify novel key anchor mutations related to small molecule interactions.

### Systematic searching for anchors in high-dimensional space

**Figure 3:**
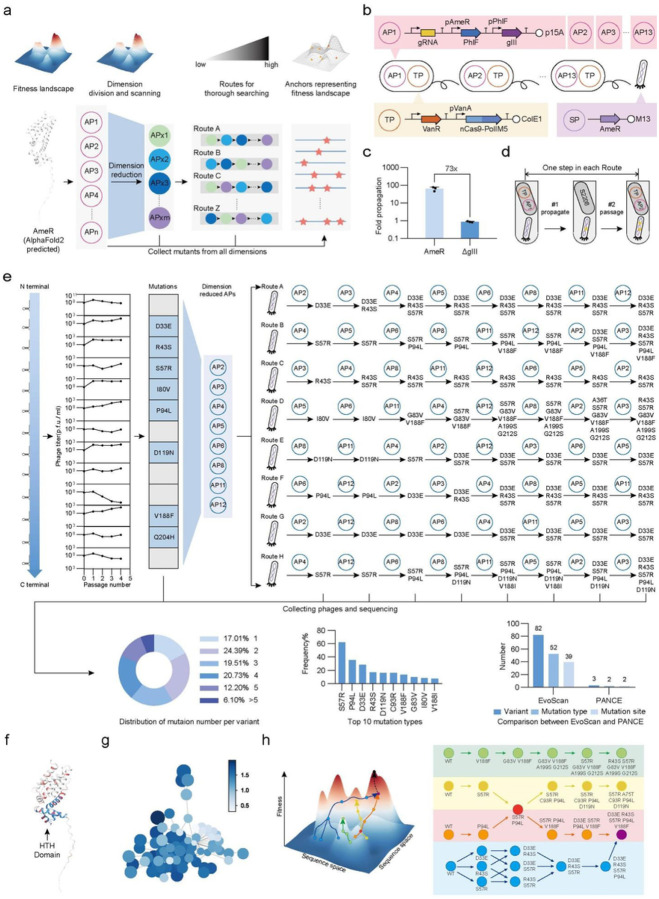
Applying EvoScan on AmeR for protein-DNA interaction evolution. (a) Schematic diagram of EvoScan of AmeR. A series of gRNAs was used to divide the AmeR gene into segments. An initial set of passages identified gRNAs that resulted in mutations, and several different evolution routes were designed with these APs. WT AmeR phages were passaged through these routes sequentially to scan and collect anchors. (b) Genetic circuit design for AmeR evolution. 13 different APs carrying 13 different gRNAs were designed. (c) Phage propagation assays of SP bearing the AmeR gene and the empty phage (ΔgIII). (d) Schematic diagram of one step in each route during evolution. (e) EvoScan of AmeR and properties of the collected variants. For each step in each route, the dominant mutations observed from supernatant were shown. Mutation number distribution and the top 10 mutation types of the 82 variants were shown. A comparison of the evolution results between EvoScan and PANCE was shown, including variant numbers, mutation diversities, and mutated sites of AmeR. (f) Distribution of mutations on AlphaFold2 predicted structure of AmeR. Red regions are mutation sites and the blue region is the typical Helix-Turn-Helix (HTH) Domain of TetR family proteins. (g) Mutation relation map among the 82 variants and WT AmeR. Each circle is a variant and its size is the mutation number. Variants are colored based on their log(fold repression). Variants with less than 3 amino acid difference were linked together. (h) Schematic and mutant information of four evolution paths from the mutation relation map. Sequences of gRNAs and genetic parts are provided in Supplementary Table 4 and Table 5. Data are mean ± SD of three experiments, except for phage titers.

Having demonstrated that EvoScan can rapidly and thoroughly explore the sequence space and generate more diverse functional variants than traditional methods, we next applied this approach to protein-nucleic acid interaction and systematically searched the space from low to high dimensions. We selected AmeR, a transcriptional regulator from the TetR family which plays important roles in many biological processes and synthetic biology^47,48^. AmeR has few known sequence homologs, making it challenging to use traditional methods to explore its sequence-function relationship, especially in high-dimensional space (Fig. 3a). We planned to first carry out a rapid scan of all gRNAs that cover the full sequence of AmeR, then select only those that generated enriched mutations for further use. Several different evolution routes could then be designed using the remaining APs. Serial passaging of phage across hosts containing different APs would identify anchors in high dimensions – that is, combining multiple mutations in different segments – that thoroughly and representatively sampled the AmeR sequence space (Fig. 3a).

To link AmeR interaction with its operator to gIII expression, we inserted a PhlF repressor after the pAmeR promoter, such that the repression ability of AmeR is positively correlated with gIII expression (Fig. 3b). We tested several combinations of plasmid origins, ribosome binding sites (RBS) and repressor types^49^ to optimize the circuit. The optimal combination resulted in 73-fold propagation of SP carrying AmeR (Fig. 3c, Extended Data Fig. 4b).

**Figure 4:**
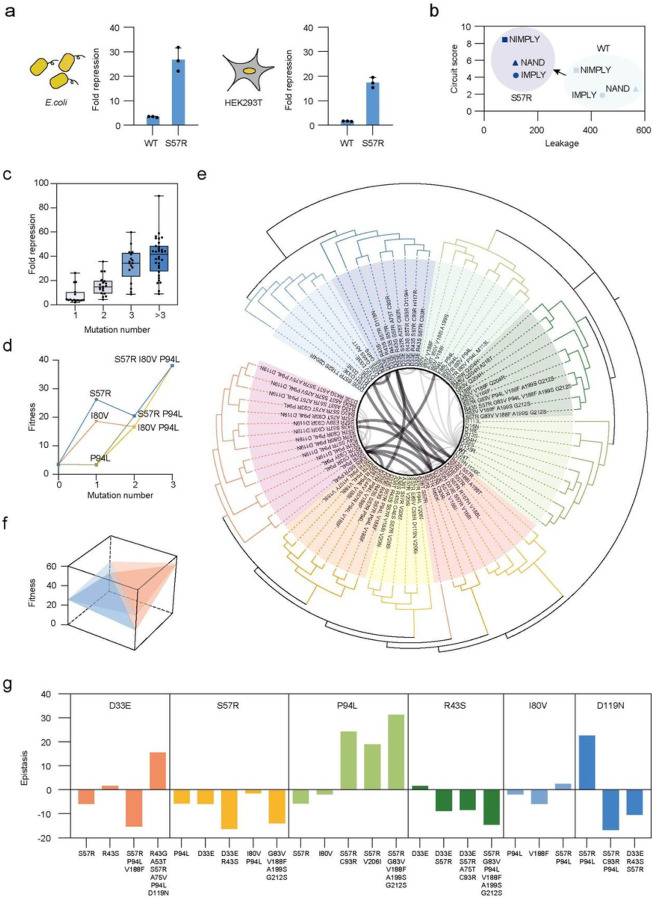
Genetic relationships and features of the 82 anchors generated by EvoScan. (a) Fold repression of WT AmeR and the S57R variant in different systems, *E. coli* and HEK293T mammalian cells. (b) Leakages and circuit scores of different genetic circuits (IMPLY, NIMPLY, and NAND) using WT AmeR or the S57R variant. (c) Fold repression of AmeR variants with different mutation numbers. (d) Evolution paths from WT to the S57R I80V P94L variant. (e) Phylogenetic tree of the 82 variants. The tree is divided into sub-regions by branch distances. Variants with less than 2 amino acid difference are linked by curves. Curves across sub-regions are in bold black. (f) Magnitude epistasis for D33E and [P94L V188F] in the S57R genetic background (upper plane), and reciprocal sign epistasis for P94L and [G83V V188F A199S G212S] in the S57R genetic background (lower plane). (g) Epistasis values of D33E, S57R, P94L, R43S, I80V, and D119N with combinations of different mutations. Data are mean ± SD of three experiments, except for phage titers.

To start the scanning process, we selected 13 gRNA sites that cover both the N-terminal and C-terminal domains of AmeR, which are involved in DNA binding and dimerization, respectively. We measured phage titers after each of the 4 passages and found that most groups enriched ≥ 50-fold. Of the 13 different groups, 8 generated dominant mutations in the phage supernatant. These mutations were observed within the targeting segment corresponding to each gRNA (Fig. 4b, 4e). These results provide one-dimensional information about the protein sequence space. We next designed 8 evolutionary routes to sample the high-dimensional space, in which SPs were passaged across all these 8 APs in different orders (Fig. 3d, 3e). For each route, we sequenced the supernatant and 2 single plaques from each round (Fig. 3e). After the full evolutionary scanning process, we obtained 82 anchor variants encompassing 52 different mutations at 39 residue sites (Fig. 3e). Among all the variants, a large portion (~ 83%) of variants had more than 2 mutations, demonstrating the successful exploration and even sampling of the high-dimensional space. We measured the fold repression of the 82 variants, and nearly all of them showed improved function compared to WT, demonstrating again the effectiveness of EvoScan in searching for high-fitness sequences (Extended Data Fig. 5a, Supplementary Table 1).

For comparison, we also applied PANCE to AmeR evolution (Extended Data Fig. 4a, 4b, 4c). After 16 passages, only R43S and S57R single mutants and the R43S S57R double mutant appeared (Fig. 3e, Extended Data Fig. 4d), all of which appeared during EvoScan within 8 passages. That only the variants from the low-dimensional space were observed in PANCE again illustrated how allowing competition between variants from all parts of sequence space can suppress and obscure many functionally informative mutation sites and high-fitness variants from the high-dimensional space, which were systematically captured by EvoScan.

### Anchors capture key features of the design space

Alignment between mutations and predicted structure by AlphaFold2 suggested that these beneficial mutations accumulated not only on the helix-turn-helix domain near the N terminus that interacts with DNA, but also on regions related to dimerization of AmeR near the C terminus (Fig. 3f). To investigate the mutation relationship between variants, we drew a relation map linking variants that contained less than three different residues (Fig. 3g). The evolution paths leading to different mutants were connected with complexity, indicating the complex interactive nature of protein evolution in high-dimensional space.

We were able to identify four evolution paths from the complex map, leading to different mutants that shared the same intermediates or reached the same destination (Fig. 3h). This suggested the existence of shared local peaks in the landscape, consistent with a recent study demonstrating the simultaneous accessibility of multiple peaks during evolution^27^. These mutations usually contained one or more of D33E, R43S, S57R, P94L, and variants containing these mutations appeared to be fitter than WT AmeR (Fig. 3g, Extended Data Fig. 5a), indicating that these mutation sites provide important information about the sequence-function relationships for high-fitness genotypes.

The best-performing single mutant, S57R, outperformed the wild-type AmeR repression ability in both bacteria and mammalian cell systems (Fig. 4a, Extended Data Fig. 6a, 6b). Repressors with better properties are crucial for robust gates and genetic circuits construction in synthetic biology (e.g., low leakage, high circuit score)^50^. We next incorporated it into several genetic circuit contexts such as IMPLY, NIMPLY, and NAND^49^ (Fig. 4b, Extended Data Fig. 6c-h). The S57R variant significantly increased the circuit score of all these genetic circuits and reduced the circuit leakage at the same time (Fig. 4b). These results show that the identified mutations affected the protein-DNA interaction directly and captured essential features of the protein itself, rather than increasing fitness only in the context of our evolution selection.

### Anchors capture complex epistasis interactions in the high-dimensional space

We also found that, in these anchors, mutational combinations had synergistically enhanced repression abilities in both *E. coli* and HEK293T, demonstrating that exploring the higher dimensions is vital for identifying proteins with improved functions (Fig. 4c, Extended Data Fig. 6a, 6b). However, we found that the order of introducing mutations significantly affected the evolvability, even if the start point and end point were the same (Fig. 4d). For example, S57R P94L double-mutants had lower fitness than S57R, which suggested that it was more difficult for natural evolution to reach the final genotype (I80V P94L S57R) if S57R was introduced first (Fig. 4d). We further built a phylogenetic tree to investigate the evolvability among these variants (Fig. 4e). The results revealed that, by designing different routes (Fig. 3e), EvoScan likely bypassed these evolvability limitations and achieved long genetic distance searching to obtain these anchors by “jumping” between domains in different orders (APs) in the high-dimensional space. These results further highlighted the need for high-throughput targeting methods to effectively explore the sequence space.

These non-additive interactions between two or more mutations are known as epistasis, which has profound impacts on the landscape in the high-dimensional space. We next systematically investigated the epistasis effect in these anchors and calculated the epistasis value (ε) using fold repression as the fitness value of different genotypes (Supplementary Table 2). We identified both negative (such as D33E and S57R, R43S and [D33E S57R A75T C93R]) and positive epistasis (such as [S57R P94L] and V188F, [P94L S57R] and [G83V V188F A199S G212S]) for different mutation combinations in both low dimensions and high dimensions (Supplementary Table 2). We also studied the magnitude and sign epistasis of different mutations (Fig. 4f), which can create rugged fitness landscapes. Interestingly, we identified reciprocal sign epistasis in the high-dimensional space, such as P94L and [G83V V188F A199S G212S] in the S57R genetic background (Fig. 4f). We also found that, even for the same mutation, such as D33E, P94L, and D119N, ε can be either positive or negative when combined with different mutations, indicating the complex and idiosyncratic epistasis relationship between different mutations (Fig. 4g).

### EvoAI enables sequence space reconstruction and prediction of new proteins

Given the complex interaction of mutations in the high-dimensional space, we next aimed to use deep learning to extract the latent features of these anchors obtained from EvoScan to represent and reconstruct the design space of AmeR for high-fitness genotypes with high accuracy, enabling design of new proteins with multiple mutations not represented in the experimental outcomes. We name this hybrid experimental-computational method EvoAI.

**Figure 5:**
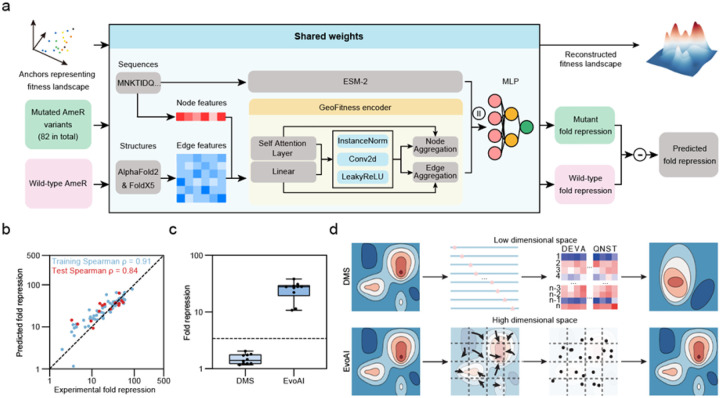
Anchors and deep learning reconstruct the design space for high-fitness genotypes. (a) Schematic of the deep learning model. WT AmeR and 82 anchors from EvoScan were the data set for model training. (b) Training and test results of the 82 variants. Training data are in blue and test data are in red. (c) Experimental fold repression of the designed variants using model trained by EvoScan anchors or deep mutational scanning (DMS) information. The dashed line is WT AmeR fold repression. (d) Comparison between DMS and EvoAI. DMS can only search the low-dimensional space, while EvoScan comprehensively segments and scans the high-dimensional space to enable accurate design space reconstruction by deep learning.

We combined a pre-trained GeoFitness model and the Protein Language Model (ESM-2), followed by a Multi-Layer Perceptron (MLP) to enhance the accuracy of predicting protein mutation effects (Fig. 5a). The pre-trained GeoFitness model was trained on a large dataset of ~ 300,000 protein fitness values from various experimental cases and indicators to enable prediction of protein fitness of single mutations (Extended Data Fig. 7). We used the 82 anchor points for both training and validation with a 10-fold cross-validation approach to obtain the final model (Extended Data Fig. 8). Spearman correlation coefficients were 0.91 and 0.84 for the training set and the test set, respectively, demonstrating a high level of consistency in training effectiveness (Fig. 5b). These results demonstrated that our deep learning model accurately predicted the multi-interaction of mutations and complex epistasis in higher dimensional space.

We further validated the accuracy of the reconstructed space by designing, predicting, and testing new variants different from the 82 anchors. To reduce the computational load, we chose 13 mutations from the top 11 mutation sites with high prediction certainties for novel protein design (Extended Data Fig. 9b). We then computationally traversed all possible combinations of 6 total mutations and calculated the predicted fold repression by our model (1093 predictions in total). The 10 top-scoring protein sequences were cloned and experimentally tested for their fold repression. All 10 sequences showed significantly improved activities compared to WT with 10- to 38-fold repression abilities (Supplementary Table 3). Furthermore, although we chose only the top predictions and all of them have very close prediction scores, these variants still showed a high Spearman correlation coefficient between prediction scores and experimental results (Extended Data Fig. 9c, 9d). For comparison, we tested the predicted sequence space without using these anchors information but only using low-dimensional deep mutational scanning (DMS) information, and also generated 10 variants with 6 mutations each (Extended Data Fig. 9e). In striking contrast to the high-performing EvoAI-predicted variants, all 10 variants generated by DMS had worse activity relative to wild-type AmeR (Fig. 5c, Supplementary Table 3).

These results validated that, with these compressed anchors, our deep learning model can accurately reconstruct the design space for high-fitness genotypes in high-dimensional space, and design new protein sequences with improved functions. We identified 39 mutation sites in AmeR (Fig. 3e) that could potentially generate high-fitness genotypes, with a theoretical design space of ~ 10^50^ (20^39^). Our EvoAI approach therefore effectively demonstrated that this vast design space of AmeR for high-fitness genotypes can be compressed by ~ 10^48^ times to 82 anchor points.

## Discussion

Navigating the complexity and scope of a protein fitness landscape is a long-standing challenge for protein design. We developed EvoScan, a novel system that combines EvolvR mutagenesis and phage selection to explore the protein sequence space in different dimensions. EvoScan can identify valuable anchors, which are variants with critical mutations that represent the sequence space. We showed that these anchor points can accurately reconstruct the space and design new proteins when coupled to deep learning methods (EvoAI), demonstrating the extreme compressibility of this space. Previous methods did not capture this insight likely because they only explored either the low-dimensional space by measuring single or double mutations, or a small region of the sequence space by saturating mutations. These methods thus might not capture the whole picture, especially the high-dimensional space (Fig. 5d).

Our approach has several important advantages over existing methods. First, it balances realistic fitness optimization and even sampling of sequence space, which can rapidly explore high dimensions and generate more diverse and functional variants, and provide richer information about sequence-function relationships. Second, by integrating empirical evolutionary scanning and deep learning models in EvoAI, we can leverage the strengths of both approaches. We could use the properties learned by deep learning to dynamically guide the scanning process. Future advances of explainable deep learning could uncover the underlying rules or patterns, and provide insights into how proteins adapt and overcome evolutionary constraints or trade-offs. Third, our method can evolve and investigate proteins that lack structural information, or that involve challenging interactions. We showed that EvoScan can capture anchors for proteins with diverse functions, such as protein-protein, protein-ligand, and protein-nucleic acid interactions. Our approach should be compatible with any biomolecular function that can be coupled to a transcriptional output (e.g., enzymes through small molecule sensors), and thus could be applied to study the sequence spaces of diverse biomolecules.

Our approach could be further improved in the future. We could use Cas9 variants with more PAM options to increase the guide RNA tiling and mutation-targeted segment selection. We could also modify the editing system to introduce mutations at multiple sites at once, avoiding host switching and speeding up the exploration process. Furthermore, incorporation of the target mutagenesis approach of EvoScan into PACE could potentially lead to deeper sampling of sequence space segments. In addition, integration of EvoScan with genotype reconstruction methods, such as Evoracle, could enable more systematic and intelligent exploration of the sequence space^26^. Moreover, the modularity of our system makes it highly suitable for automation, such as with the recently reported PRANCE method^51^, and could be scaled up to provide more comprehensive fitness landscape profiling data for different protein targets, illustrating whether the extreme compressibility of the design space for high-fitness genotypes is universal or unusual, or if the whole protein fitness landscape is compressible.

We also hope that our method will inspire new insights into the relationship between genotype and phenotype and the evolution of biological systems. The compressibility of the design space may suggest that nature somehow finds a way to search through the seemingly infinite space in the relatively short period of life time on earth by Darwinian evolution, possibly by “jumping” between these anchors instead of searching every possibility (Fig. 5d). Genetic recombination in large sexual populations could possibly enable this “jumping” and boost evolution rates^53,54^. Our approach would enable the investigation of such path dependence of evolutionary outcomes of biological systems in high-throughput experiments^51,52^ and provide valuable insights for evolution and protein design in biotechnology and biomedical applications.

## Materials and Methods

### General methods.

The following working concentrations of antibiotics were used: carbenicillin (Solarbio, 50 μg/ml), kanamycin (Solarbio, 50 μg/ml), spectinomycin (Macklin, 50 μg/ml), chloramphenicol (Macklin, 25 μg/ml). PHANTA 2x mix (Vazyme) was used for cloning PCR, and Flash 2x mix (Vazyme) was used for verification PCR and Sanger sequencing (Tsingke Bioscience). All cloning fragments were assembled by Golden Gate assembly (New England Biolabs) or ClonExpress assembly (Vazyme) methods. Plasmids were cloned in DH5α competent cells (HT Health). Synthetic genes were ordered from Tsingke Bioscience. Cloned plasmids were extracted by Tiangen DNA extraction kit. *E. coli* strain S2060^55^ was used in all aspects of the EvoScan process, including system construction, evolution, and plaque assays. The DH5α strain was used for flow cytometry experiments. Detailed information on the plasmids and selection phage (SP) used in this work is given in Supplementary Table 6.

### Phage propagation assay.

Competent S2060 cells were transformed with corresponding accessory plasmid (AP) in each experiment. Overnight cultures of single colonies inoculated in LB medium with proper antibiotics were diluted 50 or 100 times and grown at 37 °C in 220 rpm shaker (ZQZY-B8, cultured in shake tubes, 5 ml system) or 1000 rpm shaker (HUXI HW-400TG, cultured in 96-deep well plate, 500 μl system) to log phase (OD_600_ ~ 0.4–0.6). These cells were then infected with SP at an initial titer of 5 × 10^6^ plaque-forming units (p.f.u.) per ml. The mixture was further cultured overnight (16–20 h) at 37 °C in the shakers as described above, and was centrifuged at 4000 rpm for 10 min. Phages in the supernatant was filtered by 0.22 μm bacterial filter and stored at 4 °C for further use.

### Plaque assay.

A single colony of chemically competent S2208 cells^55^ (S2060 cells transformed with plasmid pJC175e) was cultured overnight in LB medium added with proper antibiotics. The saturated bacteria culture was diluted 50 or 100 times into LB medium with proper antibiotics and grown at 37 °C in 220 rpm shaker to log phase (OD ~0.4–0.8) before use. Phages were serially diluted 6 to 8 times with a dilution ratio of 10-fold in each step in LB medium. Then, 10 μl of each phage dilution was mixed with 45 μl S2208 cells, and then 180 μl of liquid (50–65°C) soft agar (LB medium and 0.5% agar) supplemented with 2% Bluo-gal (Inalco S.p.A.) was added and mixed by pipetting. The whole mixture was immediately added onto 500 μl of bottom agar (LB medium and 1.5% agar) previously prepared in 24-well plate. Then the plates were incubated in 37 °C for overnight growth (14–18 h).

### Calculation of fold propagation.

For fold propagation measurement of the selection phage, initial phage titer and final phage titer were measured by plaque assays. We defined the ratio of final phage titer versus initial phage titer as the fold propagation of the phage.

### Basic process of evolutionary scanning (EvoScan).

Target mutagenesis plasmid (TP) was first transformed to chemically competent S2060 cells, and then the prepared S2060-TP cells were used to prepare super chemically competent cell by Inoue method^56^. Chemically competent S2060-TP cells were transformed with corresponding APs. The resulting S2060-TP-AP bacteria were cultured overnight and diluted 50–100 times into 500 μl LB medium with antibiotics and inducers, and grown in 37 °C 1000 rpm shaker to OD ~0.5. The phage titer for the first infection was around 5×10^6^–5×10^8^ p.f.u./ml, and for the following passages the phages were subjected to a 1:50 or 1:100 dilution. Vanillic acid (Sigma-Aldrich, ethanol dissolved) at a final concentration of 50 μM was added to induce the expression of nCas9-PolIM5 complex. The mixture was then cultured in 37 °C 1000 rpm shaker overnight. The next day the mixture was centrifuged at 4000 rpm for 10 min and the phage content of the collected supernatant was verified by PCR (Flash 2x mix) and Sanger Sequencing. The supernatant was then used for plaque assay as described above. Single plaques from plaque assay were picked and further verified by PCR (Flash 2x mix). The PCR product was sent for Sanger Sequencing.

### Searching steps in each route.

For each step of EvoScan in a route, 10 μl supernatant with evolved phages was added into 1 ml log-phase S2208 bacteria culture (OD ~0.4–0.8), and propagated overnight in 96-deep well plate. The mixture was centrifuged at 4000 rpm for 10 min and filtered by 0.22 μm bacterial filter. The obtained phages were then diluted and infected another host cell containing a different AP with an infection titer of 5×10^6^ p.f.u./ml.

### Basic process of phage-assisted non-continuous evolution (PANCE).

Accessory plasmid with the designed genetic circuit and the mutagenesis plasmid MP6 were co-transformed into super chemically competent S2060 cells. The S2060-MP6-AP bacteria were cultured overnight and diluted 50–100 times into 500 μl LB medium with antibiotics and inducers in 96-deep well plate, and grown in 37 °C 1000 rpm shaker to OD ~0.5. The initial phage titer was around 5×10^6^–5×10^8^ p.f.u./ml, and the phages were subjected to a 1:10–1:100 dilution in the following passages in a 500 μl system. 1% (m/v) arabinose dissolved in ddH_2_O was added as the inducer of MP6. Phages were then collected to obtain mutations following the same procedures in EvoScan.

### Induced expression assay.

Single colonies of strains to be tested were cultured in LB medium overnight. Saturated bacterial culture was diluted 100 times in LB medium with proper antibiotics and inducers, and cultured in 37 °C 1000 rpm shaker for 2 h (OD ~ 0.4). Then LB with proper antibiotics and inducers was prepared and 2 μl log phase bacteria culture was added together to a whole volume of 500 μl. The mixture was cultured for 5 hours in the 96-deep well plate.

### Flow cytometry assay.

10 μl of the culture was added into 190 μl PBS with 2 g/L kanamycin in the 96-well U-bottom plate to stop the cell growth. The plate was stored in 4 °C until used. The flow cytometer (Beckman Coulter Cytoflex S) was used to quantify the expression levels of fluorescent protein. The software FlowJo v10 was used to gate the events (at least 10000 events) and calculated the median of each sample.

### M^pro^ drug resistance index.

In the RTHS protease activity assay, the fluorescence of the experimental group carrying eYFP was measured with or without addition of M^pro^ inhibitor GC376 or PF-07321332. The ratio of fluorescence FITC-A median with inhibitor versus fluorescence FITC-A median without inhibitor was defined as the resistance index (RI) to evaluate the drug resistance abilities of different M^pro^ variants.

### Structure display and interaction prediction.

Schrodinger 2017 was used for structural display. ZDOCK^57^ was used for interaction structure prediction between EGFP and its nanobody. The interaction between M^pro^ and inhibitors within 3 angstrom was shown in the figure.

### Fold repression calculation.

The background fluorescence of cells, which is the median of the fluorescence of the bacteria carrying an empty plasmid with only the backbone, was measured and subtracted from all the experimental groups. The subtracted fluorescence values of the uninduced group (no repressor expression) were divided by the induced group (repressor expression) to obtain the fold repression.

### Relative expression level calculation.

Using flow cytometry assay, we measured the FITC-A median of the strain carrying the empty plasmid and set this value as the background value. The FITC-A median of the strain carrying the standard plasmid expressing eYFP through the open reading frame J23101-B0064-YFP was measured the same way and set as the standard value. The FITC-A median of the strain containing a specific variant was measured the same way, and the relative expression level was defined as: (variant value – background value)/standard value.

### Circuit score calculation.

Thestrain carrying the plasmid with a specific genetic circuit was prepared for flow cytometry assay. IPTG (1 mM) and vanillic acid (100 μM) were used as the input signals. YFP was used as the output reporter of the circuit and the FITC-A median of each state was measured. The lowest ON signal (lowest FITC-A median in “ON” states of the circuit) was divided by the highest OFF signal (highest FITC-A median in “OFF” states of the circuit) to obtain the circuit score.

### AmeR phylogenetic tree construction.

Protein sequences of the 82 variants and the WT were collected as a fasta file and the file was input into MEGA11 for multiple sequence alignment (MSA)^58^. After MSA and phylogenetic analysis, neighbor-joining tree was selected as the method of tree construction. The output tree was decorated by iTOL^59^, and all the parameters were set using default values.

### Epistasis calculation.

Epistasis between two different mutations, A and B, could be calculated as ε = fab + fAB – fAb – faB. f is the fitness of wild-type, double-mutant and single-mutant genotypes, respectively. ε > 0 means positive epistasis, while ε < 0 means negative epistasis.

### Mammalian Cell culture and transfections.

HEK293T cells (CRL-3216, ATCC) were cultured in Dulbecco’s modified Eagle’s medium (DMEM, Gibco) supplemented with 10% (v/v) fetal bovine serum (FBS, Biological Industries) and 1% (v/v) penicillin/streptomycin solution (Beyotime) at 37 °C, 100% humidity and 5% CO_2_. In transfection experiments, 60,000–80,000 HEK293T cells in 0.2 ml of DMEM complete medium were seeded into each well of 48-well plastic plates (NEST) and grown for ~24 h. M5 HiPer Lipo2000 Transfection Reagent (Lipo2000, Mei5bio) was used in all transfection experiments following the manufacturer’s protocol. Briefly, a sample mixture was prepared by mixing 150 ng repressor plasmid or 150 ng control plasmid (repressor-deficiency) with 150 ng reporter plasmid in 0.7 μl Lipo2000. The mixture was incubated at room temperature for 20 min before adding to cells. Transfections were supplemented with 0.2 mL DMEM complete medium 24 h post-transfection. Cells were cultured for 2 days post-transfection before flow cytometry analysis.

### Mammalian cell flow cytometry assay.

Cells were trypsinized 48 h after transfection and were then centrifuged at 250 × g for 10 min at room temperature. The supernatant was removed, and the cells were resuspended in 1 × PBS. Fluorescence values were measured with a Cytoflex flow cytometer (Beckman Coulter, Inc.). PB450-A and ECD-A channels were chosen for BFP and mCherry measurement, respectively. Data were processed using FlowJo (TreeStar), gated by the area of the forward scatter and the side scatter (FSC-A/SSC-A) and then cell populations were selected by gating out the background BFP signal of untransfected cells to obtain the median of fluorescence. The median of fluorescence was calculated for >20,000 transfected cells for each sample. To reduce expression noises between samples, the mCherry : BFP fluorescence ratio was used to report the repressor activity^60^. The mCherry : BFP fluorescence ratio was calculated by (mCherry - mCherry_0_)/(BFP - BFP_0_), mCherry_0_ and BFP_0_ were the fluorescence values from untransfected HEK293T cells. The fold-repression was calculated by (mCherry : BFP)_unrepressed_/(mCherry : BFP)_repressed_. (mCherry : BFP)_unrepressed_ and (mCherry : BFP)_repressed_ were the fluorescence values of the states co-transfected with control plasmid or repressor plasmid.

### Feature generation.

Our initial step entails querying the UniRef30_2021_03 and bfd multiple sequence alignment (MSA) databases. Subsequently, we employ AlphaFold2 to construct the structural representation of the wild-type protein. For this endeavor, we deploy the GeoFitness-Seq variant of the pre-training model. In the case of mutated proteins, structural configurations are generated using FoldX 5. The sequence features are extracted from the large-scale protein language model ESM-2, for the purpose of capturing global context information. Consequently, each node in the Geometric Encoder is initialized by the embedding of the corresponding residue derived from the ESM-2. Unlike conventional methodologies that rely upon inter-residue distances and contacts to establish edges, each edge in the Geometric Encoder is initialized by the relative geometric relationship between a pair of residues derived from the protein 3D structure^61^.

### Cross-validation.

We employed a 10-fold cross-validation approach to find the hyperparameters of the model. The dataset, comprising 82 mutational data points, was divided into three parts: a training set (59 samples), a validation set (7 samples), and a test set (16 samples). Model evaluation was performed using the Spearman correlation coefficient (ρ) as the primary assessment metric.

### Model training details.

The model employs the Soft Rank Loss as its loss function, with a learning rate of 10^−3^, Adam optimizer, and a decay rate for the learning rate. The training spans across 50 epochs. Subsequently, the learning rate of the upstream GeoFitness model is set to 10^−4^, while the learning rate of the downstream model is adjusted to 5×10^−4^ for further ne-tuning.
